# Supplementary material for: Tunable unidirectional nonlinear emission from transition-metal-dichalcogenide metasurfaces
Source: Nat Commun. 2021 Sep 22;12:5597. doi: 10.1038/s41467-021-25717-x (PMC8458373; doi:10.1038/s41467-021-25717-x)
Supplement: Supplementary file 1 — Supplementary Information [file 41467_2021_25717_MOESM1_ESM.pdf]

Supplementary Information for

Tunable Unidirectional Nonlinear Emission from Transition-Metal-Dichalcogenide Metasurfaces

Mudassar Nauman[a,b], Jingshi Yan[b], Domenico de Ceglia[c], Mohsen Rahmani[d], Khosro Zangeneh Kamali[b], Costantino De Angelis[e], Andrey E. Miroshnichenko[f], Yuerui Lu[a] and Dragomir N. Neshev[b]

[a] *School of Engineering, Australian National University, Canberra ACT, 2601, Australia*
[b] *ARC Centre of Excellence for Transformative Meta-Optical Systems, Department of Electronic Materials Engineering, Research School of Physics, The Australian National University, Canberra ACT, 2601, Australia*
[c] *Department of Information Engineering, University of Padova, Via G. Gradenigo, 6/B, Padova, Italy*
[d] *Advanced Optics and Photonics Laboratory, Department of Engineering, School of Science and Technology, Nottingham Trent University, Nottingham, NG11 8NS, UK*
[e] *Department of Information Engineering, University of Brescia, Via Branze 38, 25123 Brescia, Italy*
[f] *School of Engineering and Information Technology, University of New South Wales, Canberra ACT, 2600, Australia*

dragomir.neshev@anu.edu.au yuerui.lu@anu.edu.au, andrey.miroshnichenko@unsw.edu.au, domenico.deceglia@unipd.it

In this work, we presented linear and nonlinear response of transition-metal-dichalcogenide (TMD) MoS_2 metasurface. Specifically, exploiting the high index of MoS_2, we observed sub-diffractive second harmonic (SH) and third harmonic (TH) response. Intriguingly, the presented MoS_2 metasurface possess the capability to switch the SH emissions in forward and backward directions.

Simulated Linear Response

We employed scanning electron microscopy to extract the parameters of the fabricated metasurfaces and name them as A, B and C, according to their periodicity and diameter of the truncated cone. The periodicity and the diameter increase as we move from A toward C. To the perform the numerical simulations, the values of refractive index and extinction coefficient, for bulk MoS_2, are extracted from the work of Beal and Hughes[1], as shown in Supplementary Figure 1.The simulated transmittance spectrum of MoS_2 metasurfaces A, B, and C is presented in Supplementary Figure 1b. Two clear transmittance dips in the SH spectrum (700-800 nm) correspond to the resonant excitation of electric dipole (ED) and magnetic dipole (MD) in each metasurface. However, two weak resonant dips, around 700-715 nm, in the linear spectrum of metasurface B and C corresponds to magnetic quadrupole (MQ). The resonance of MQ mode is weak because of absorption coefficient of MoS_2 around 700nm. The simulated transmittance spectra find qualitative agreement with the experimental results. The small discrepancy likely originates from the uncertainty of the extracted parameters, shape of the cone, and Fabry-Perot resonances present between the layered TMDs[2]. Moreover, in Supplementary Figure 1b, along with other high order Mie-resonances below 500 nm, one can observer resonance corresponding to the first order diffraction, which we call the wood's anomaly (WA).

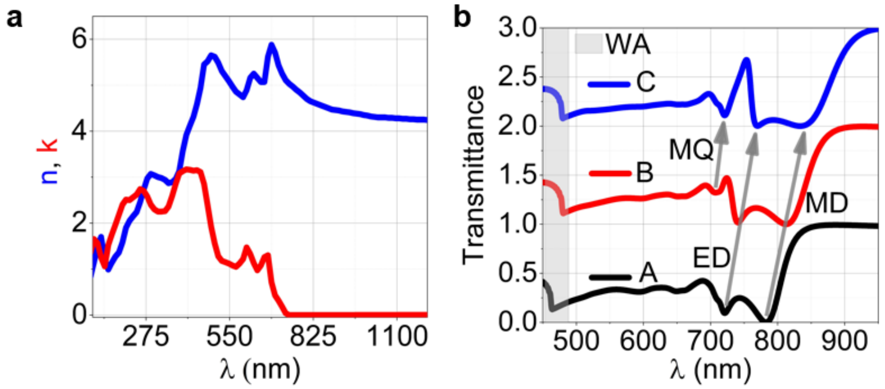

Supplementary Figure 1. Simulated linear spectrum. **a** Refractive index and extinction coefficient of Bulk MoS_2. **b** Simulated transmission spectrum of three different metasurfaces (A, B and C) having same height (around 150nm) but different radii, 100 nm, 110 nm and 120 nm, respectively. For clear understanding spectra for three different metasurfaces are vertically displaced by $T = 1$. Resonances around 500 nm are Wood's anomalies associated with the onset of the first diffraction order.

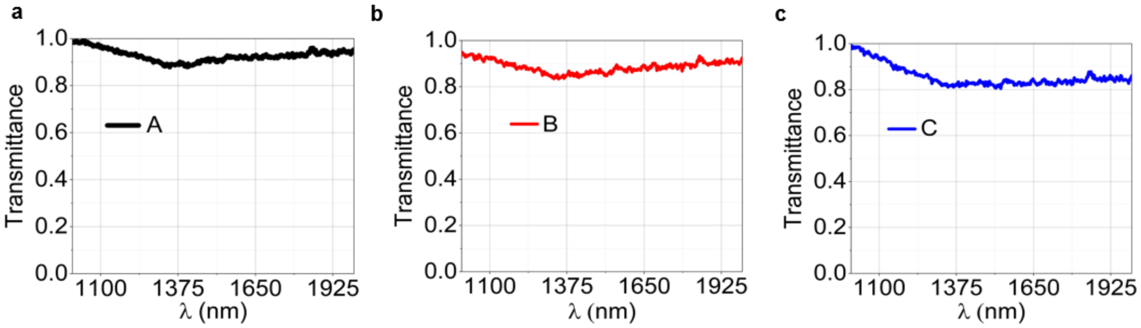

Supplementary Figure 2. Measured transmission spectra in the IR spectrum **a** metasurface A. **b** metasurface B. **c** metasurface C.

To avoid emissions of multiple diffraction orders with SH and TH emissions, we wanted our metasurfaces to be transparent in the infrared (IR) spectrum, which is the spectrum of our tunable femtosecond laser pump. All of the fabricated metasurfaces A, B, and C are transparent in the IR spectrum, which can be observed from the measured transmittance response as shown in the Supplementary Figure 2a-2c.

Third Harmonic Generation and Second Harmonic Generation Efficiency

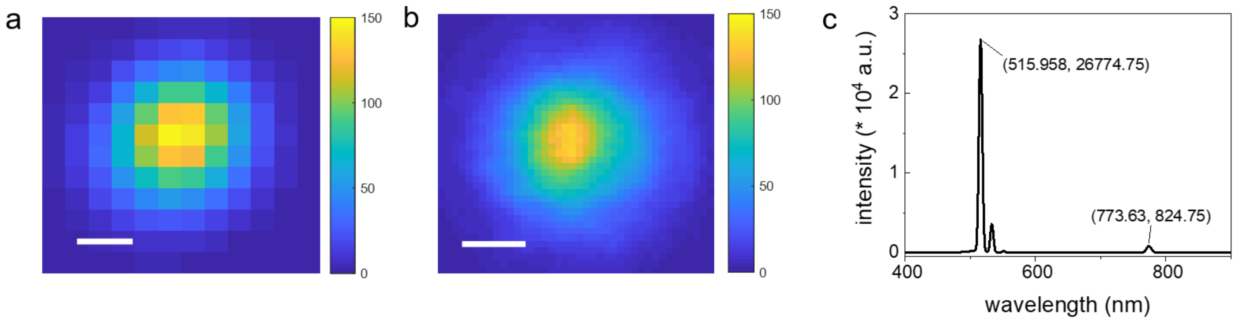

Supplementary Figure 3. THG and SHG efficiency estimation. **a** THG image from camera with 10 ms integration time. Scale bar is 1 μm. **b** calibration laser at 532 nm image from camera with 0.16 ms integration time. Scale bar is 5 μm. **c** THG and SHG spectrum by 1550 nm pump laser.

THG intensity was obtained by Thorlabs CMOS camera with 10 ms integration time, as shown in Supplementary Figure 3a. Then a 532 nm laser was used to calibrate the camera intensity to power, which was shown in Supplementary Figure 3b. After getting the ratio of camera counts to actual power from the calibration laser, we can convert the THG counts in camera to power. Therefore, by dividing the power of 1550 nm pump laser, the THG conversion efficiency is 1.01×10^{-9} at average power of 68 mW. The peak power of the laser is $P_p = \frac{P_{avg}}{f\tau} = 4.3$ kW, where $f = 80$ MHz is the repetition rate of the laser, $\tau = 200$ fs is the pulse duration. The pump beam diameter is measured to be 5.48 μm (full width at half maximum of 4.6 μm), resulting in pump peak intensity of 18.2 GW/cm^2. As SHG intensity is around 30 times weaker than THG at 1550 nm pump from the spectrum in Supplementary Figure 3c, the SHG efficiency is estimated to be around 3.4×10^{-11} with the same pump power. From the wavelength dependence Figure 5a of the main text, SHG efficiency can be increased by 10 times when tuning the pump wavelength to 1400 nm, reaching maximum SHG efficiency of 3.4×10^{-10}.

Simulated Third Harmonic Response

We perform full wave nonlinear simulations to calculate the wavelength dependent THG efficiency of the metasurfaces A and B, as shown in the Supplementary Figure 4a and Supplementary Figure 4b, respectively. The grey shading region represents the WA, which is the function of periodicity and substrate index, and divide the TH response into diffractive (below the WA) and non-diffractive (above the WA) regimes. Additionally, the position of the WA is different because both metasurfaces have the different periodicity. Both metasurfaces show enhanced TH efficiency in the nondiffractive regime of (1600-1700 nm). However, the THG efficiency of both metasurfaces drastically reduce, below the WA, in the diffractive regime. Except the WA, no other strong features or resonances observed in the THG efficiency of MoS_2 metasurface.

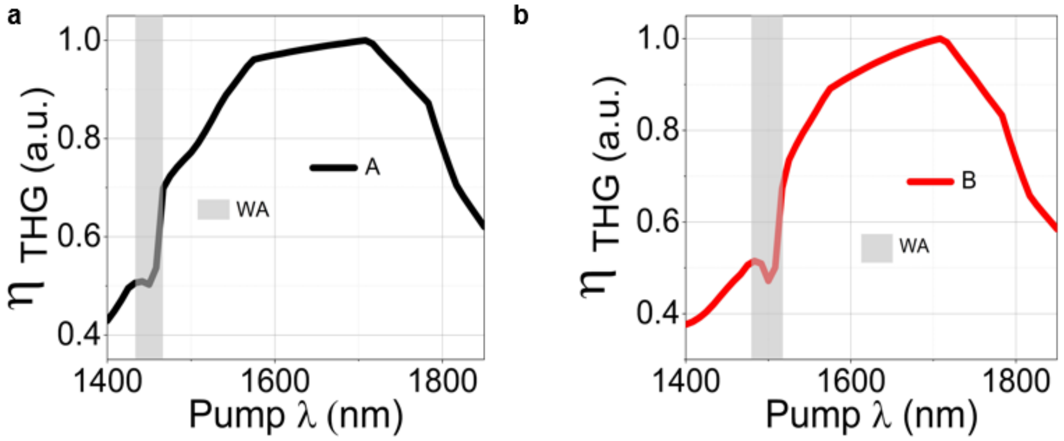

Supplementary Figure 4. Third harmonic conversion efficiency calculated with full wave nonlinear simulations. **a** Metasurface A. **b** Metasurface B.

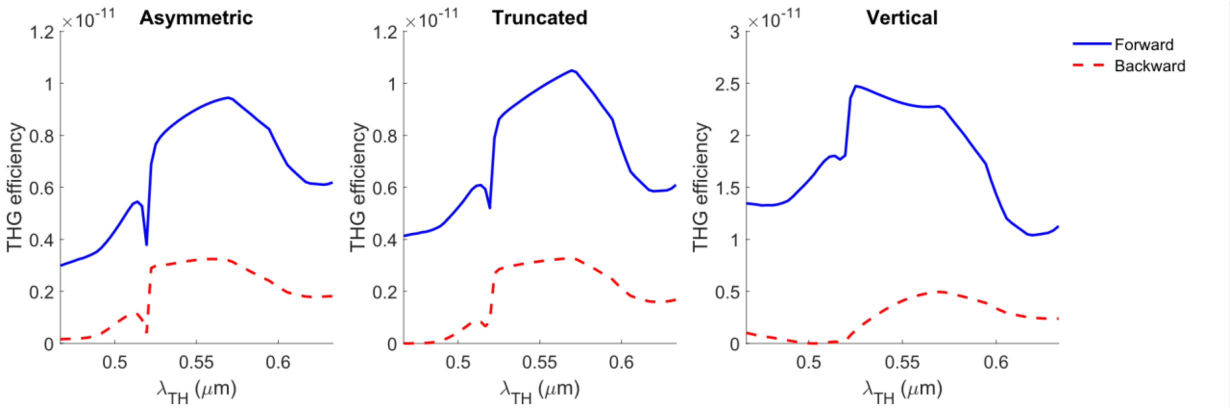

Supplementary Figure 5. Tapering angle effect on the THG conversion efficiency in either direction forward and backward, (left) asymmetric cone, (middle) truncated cone, and (right) vertical cylinder.

Simulated Second Harmonic Response

The intensity and the position of SHG peak strongly depends upon the modal overlap between the SH current source and induced mode eigenfields. Hence, the exact knowledge of the location of the second order nonlinear current source in the meta-atom is very important. Therefore, to find out the location, we performed full wave nonlinear simulations and calculated the SH conversion efficiency for four different scenarios: origin of the second order nonlinearity is at (i) top and bottom surfaces (shown in the main text), (ii) bottom surface only, Supplementary Figure 6a, (iii) top surface only, Supplementary Figure 6b, and (iv) bulk, Supplementary Figure 6c. MoS_2 metasurface possess three induced geometrical modes known as MQ, ED and MD in the SH spectrum (700-800 nm). Indeed, the three peaks in the SHG efficiency around 1420 nm, 1500 nm and 1750 nm, in all scenarios, are due to the coupling of the SH impressed source with the MQ, ED and MD modes of the metamolecule, respectively. If the origin of SH light is at the bottom surface only, as depicted in Supplementary Figure 6a, then the coupling of the SH light with MQ and MD modes is quite optimal, showing strong SH peaks. However, weak SH peak around 1500 nm is due to the poor overlap of SH source with ED eigenfields. The same physics lies behind the different intensity of SH peaks at 1420 nm, 1500 nm and 1750 nm, in other two scenarios.

SHG Efficiency of Vertical Cylinder and Asymmetric Conical Metamolecule

We employed quasinormal modes (QNMs) theory to calculate the SHG efficiency for three different shapes. The dimensions are presented in Supplementary Table1. All other dimensions like height (150 nm) and pitch (300 nm) is same.

Supplementary Table 1. Dimensions of the meta-atoms

Shape of Meta-atom	Size of top face	Size of bottom face
Asymmetric cone	90 nm	240 nm
Truncated cone	150 nm	240 nm
Vertical cylinder	240 nm	240 nm

The calculated SHG efficiency, with QNMs theory, of truncated cone meta-atom (our fabricated meta-atoms) is presented in the main text. Whilst, the SHG efficiency of asymmetric cone and vertical cylinder is presented in the Supplementary Figure 7a and Supplementary Figure 7b respectively. The figures show the calculated SH efficiency of each individual geometrical mode and their contribution toward the total SHG efficiency. It may be observed in Supplementary Figure 7a, in case of asymmetric cone, each individual mode like MQ, ED, and MD shows peaks of SH efficiency around 700 nm, 725 nm, and 800 nm respectively. The contribution of each mode toward total SH efficiency is almost identical, shown by black line in the Supplementary Figure 7a. However, in case of cylinder, magnetic modes contribute strongly towards total SH efficiency, as illustrated in Supplementary Figure 7b. Whilst, the contribution of electric modes toward total SH efficiency, just appeared as shoulder around 815 nm, shown by black line in Supplementary Figure 7b. The reason behind is that magnetic modes significantly inhibit the coupling of SH light with ED mode. In a nutshell, the cylinder shows best performance as compared to other shapes.

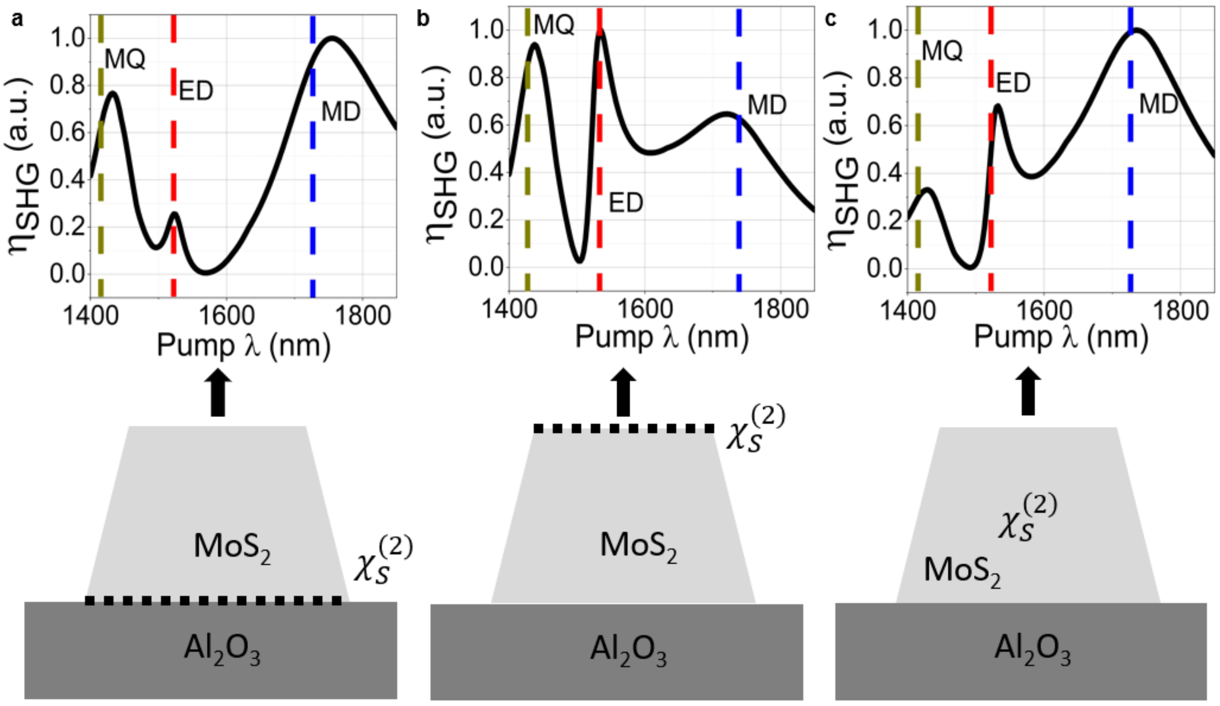

Supplementary Figure 6. Location of nonlinearity source in MoS_2 truncated cone meta-atom. **a** source location: bottom surface. **b** source location: top surface. **c** source location: bulk.

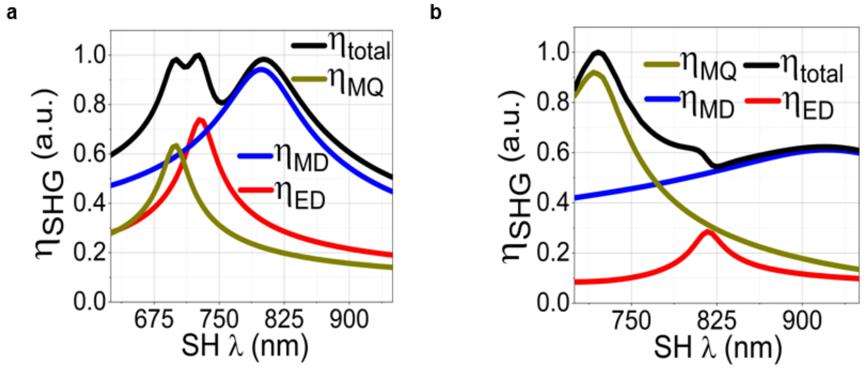

Supplementary Figure 7. SHG conversion efficiency calculated with quasi-normal modes theory. **a** Asymmetric cone. **b** Vertical straight cylinder.

Simulated Polarization Resolved Second Harmonic Response

We calculated the polarization dependent SH response of the presented MoS_2 metasurface in the forward direction (shown in the main text) and backward direction, as illustrated in Supplementary Figure 8. Polarization dependent SHG is function of two angles, $\boldsymbol{\phi_a}$ (angle between armchair direction and lab frame axis) and $\boldsymbol{\phi_p}$ (angle between pump polarization and lab frame). In the simulations, we fixed the $\boldsymbol{\phi_a = 0}$ and simulated SH efficiency as function of pump wavelength and pump polarization, as depicted in Supplementary Figure 8a. The mapping shows three peaks around 1420 nm, 1560 nm and 1750 nm. These peaks are associated with resonant excitation of the three modes MQ, ED and MD respectively.

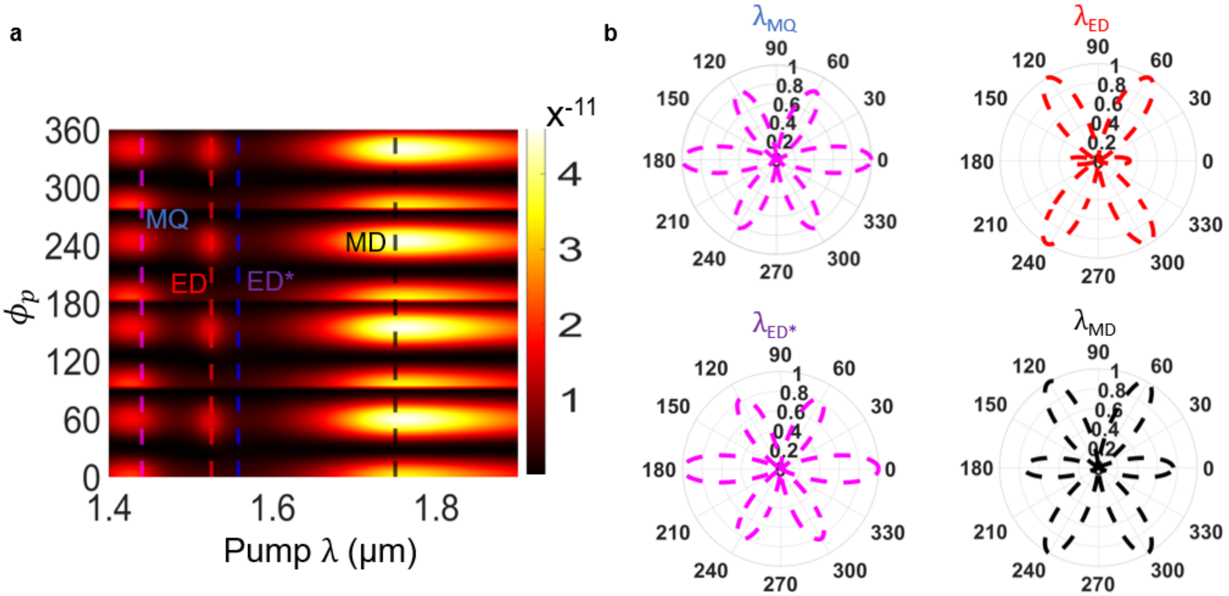

Supplementary Figure 8. Simulated polarization resolved SHG in backward direction. **a** SHG mapping as function of $\emptyset_p$ (when $\emptyset_a$= 0) and pump wavelength. **b** Polarization resolved SHG at and/or around MQ, ED, ED* and MD, where ED* is the point in close vicinity of ED, as shown in the mapping.

In Supplementary Figure 8b, the polar plots titled λ_{MQ}, λ_{ED} and λ_{MD} represents the extracted SH conversion efficiency at three photonic modes i.e. MQ, ED and MD, as function of pump polarization when $\boldsymbol{\phi_a = 0}$. Whilst, the λ_{ED^*} is the calculated SH conversion efficiency in the vicinity of ED mode as indicated by ED* in Supplementary Figure 8a. In case of magnetic modes, the coupling of the SH light with MQ and MD is quite optimal. Therefore, only one mode radiates the SH light and we receive the six SH peaks of equal amplitude. However, because of the interference of the

magnetic modes at ED and ED*, we receive four stronger and two weaker SH peaks and two stronger and four weaker SH peaks, respectively.

Additionally, the polarization resolved SH response for asymmetric cone in the forward and backward directions is depicted in Supplementary Figure 9a and Supplementary Figure 9b respectively. Whilst, Supplementary Figure 9c and Supplementary Figure 9d illustrate the polarization dependent SHG efficiency of the cylindrical metamolecule in the forward and backward direction respectively. In case of cylinder the coupling of the SH light is quite optimal.

Effect of Shape and Symmetry of Metamolecule on SHG

The shape and symmetry of metamolecule strongly affects the shape of polarization resolved SHG. For fully asymmetric cone, Supplementary Figure 9a and Supplementary Figure 9b depict the simulated SHG as function of pump polarization and wavelength in the forward and backward directions respectively.

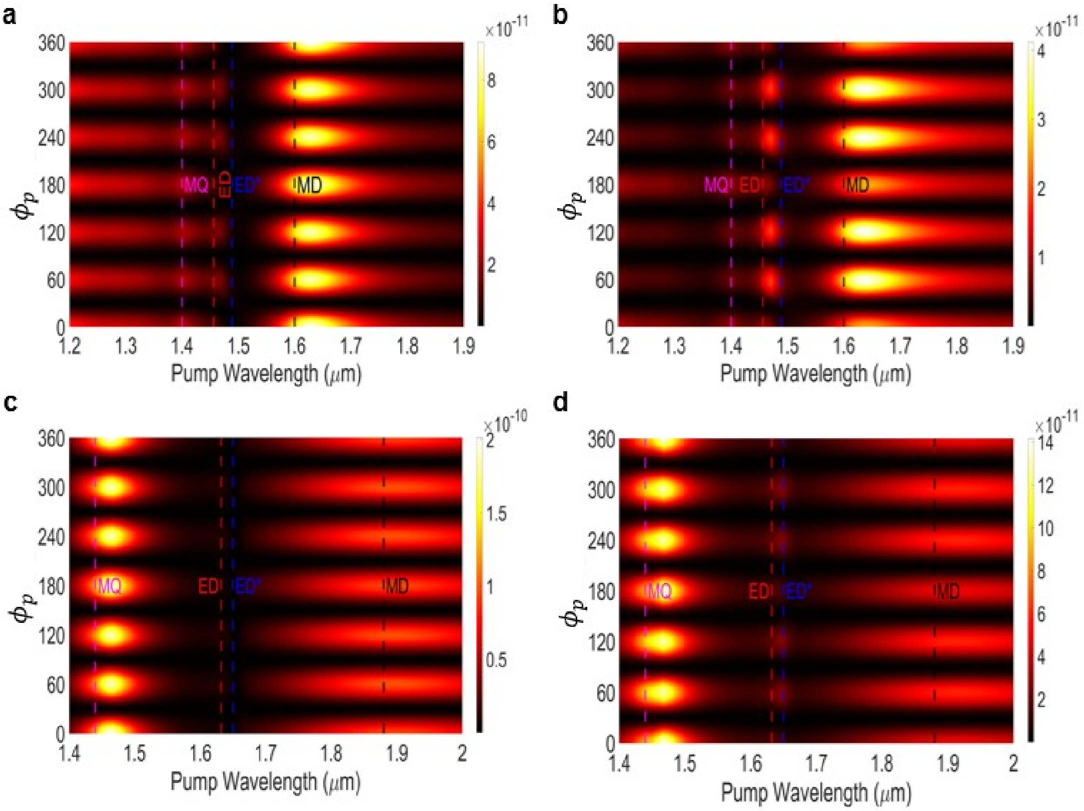

Supplementary Figure 9. Simulated SHG mapping, as function of $\emptyset_p$ (when $\emptyset_a$= 0) and pump wavelength. **a** Forward direction: Asymmetric cone. **b** Backward direction: Asymmetric cone. **c** Forward direction: vertical straight cylinder. **d** Backward direction: vertical straight cylinder.

It can be observed, in either direction, the response of the MD mode around 1600 nm, is quite strong in comparison to the MQ and ED modes. However, the contribution of MQ and ED modes is also visible in either direction around 1400 nm or 1500 nm respectively. For vertical cylinder meta-atoms, Supplementary Figure 9c and Supplementary Figure 9d illustrate the simulated SHG efficiency in forward and backward directions. The SH efficiency calculated as function of pump wavelength and its polarization. In case of cylinder, in comparison to asymmetric cone, the shape and symmetry has been changed. Hence, a clear shift in the positions and intensity of the SH emissions can be observed in Supplementary Figure 9c and Supplementary Figure 9d. When the shape and symmetry of the metamolecule change from fully asymmetric cone to vertical cylinder, following changes can be observed. The SH emissions, corresponding to MQ, ED and MD modes, undergoes red shift in the spectrum. Additionally, the interference among MQ, ED and MD modes changes the intensity of the SH emissions. For vertical cylinder, the magnetic modes become dominant

and the interference among magnetic modes suppress or inhibit the contribution of electric mode in either direction (forward and backward), as shown in Supplementary Figure 9c and Supplementary Figure 9d.

Second Harmonic Generation Directionality

The capability of the MoS_2 metsurface to switch the SH emissions in forward and backward directions influenced by the following factors, (i) interference of the induced and electric and magnetic modes, (ii) intrinsic property of MoS_2, and (iii) location of the second order nonlinear tensor. We extracted the SHG directionality by taking the ratio of SH emissions in the forward and backward directions and plotted against the pump wavelength and its polarization for different shapes, (i) truncated cone and vertical cylinder meta-atoms (shown in the main text) and (ii) fully asymmetric cone, as shown in the Supplementary Figure 10. The switching of SHG directionality can be observed around 1480 nm, as function of pump polarization. Along with modes interference, this feature strongly depends upon the shape and symmetry of the meta-atoms as well. Among three different shapes i.e truncated cone, fully asymmetric cone, and vertical cylinder, the metasurface consists of vertical cylinder meta-atoms shows best performance and acts as virtual mirror for SH light.

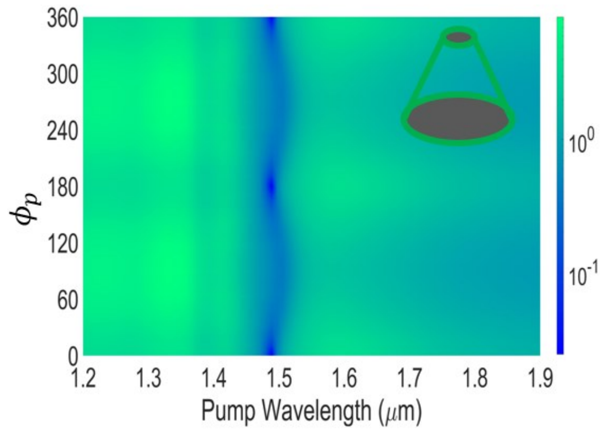

Supplementary Figure 10. Simulated SHG directionality, as function of $\emptyset_p$ (when $\emptyset_a$= 0) and pump wavelength: Fully asymmetric cone.

Supplementary References:

1. Beal, A. R. & Hughes, H. P. Kramers-Kronig analysis of the reflectivity spectra of 2H-MoS2, 2H-MoSe2 and 2H-MoTe2. *J. Phys. C Solid State Phys.,* **12**, 5, 881-890, 1979.

2. Munkhbat, B. *et al.* Self-Hybridized Exciton-Polaritons in Multilayers of Transition Metal Dichalcogenides for Efficient Light Absorption. *ACS Photonics,* **6**, 1, 139-147, 2019.

Supplementary Table 2. MoS_2 Dispersion Data

WL	n	k	WL	n	k	WL	n	k
8.11E-02	7.40E-01	1.16E+00	5.36E-01	5.20E+00	1.15E+00	1.00E+00	4.34E+00	6.11E-04
9.28E-02	8.37E-01	1.36E+00	5.48E-01	5.09E+00	1.12E+00	1.01E+00	4.32E+00	7.74E-04
1.04E-01	1.15E+00	1.65E+00	5.59E-01	4.97E+00	1.08E+00	1.03E+00	4.31E+00	9.38E-04
1.16E-01	1.51E+00	1.56E+00	5.71E-01	4.86E+00	1.05E+00	1.04E+00	4.30E+00	1.10E-03
1.28E-01	1.70E+00	1.25E+00	5.83E-01	4.80E+00	1.12E+00	1.05E+00	4.30E+00	1.27E-03
1.39E-01	1.21E+00	1.06E+00	5.94E-01	4.74E+00	1.22E+00	1.06E+00	4.30E+00	1.43E-03
1.51E-01	9.90E-01	1.52E+00	6.06E-01	4.89E+00	1.47E+00	1.07E+00	4.29E+00	1.60E-03
1.63E-01	1.14E+00	1.75E+00	6.18E-01	5.16E+00	1.33E+00	1.08E+00	4.29E+00	1.76E-03
1.74E-01	1.27E+00	1.96E+00	6.29E-01	5.25E+00	1.13E+00	1.10E+00	4.28E+00	1.93E-03
1.86E-01	1.36E+00	2.18E+00	6.41E-01	5.16E+00	9.75E-01	1.11E+00	4.28E+00	2.09E-03
1.98E-01	1.58E+00	2.40E+00	6.53E-01	5.07E+00	1.08E+00	1.12E+00	4.27E+00	2.26E-03
2.09E-01	1.90E+00	2.51E+00	6.64E-01	5.07E+00	1.26E+00	1.13E+00	4.27E+00	2.43E-03
2.21E-01	2.05E+00	2.50E+00	6.76E-01	5.62E+00	1.30E+00	1.14E+00	4.26E+00	2.59E-03
2.33E-01	2.15E+00	2.56E+00	6.88E-01	5.88E+00	7.12E-01	1.15E+00	4.26E+00	2.76E-03
2.44E-01	2.24E+00	2.62E+00	6.99E-01	5.71E+00	4.30E-01	1.17E+00	4.25E+00	2.93E-03
2.56E-01	2.46E+00	2.74E+00	7.11E-01	5.43E+00	3.14E-01	1.18E+00	4.25E+00	3.09E-03
2.68E-01	2.73E+00	2.70E+00	7.23E-01	5.25E+00	1.82E-01	1.19E+00	4.25E+00	3.26E-03
2.79E-01	2.97E+00	2.58E+00	7.34E-01	5.11E+00	3.91E-02	1.20E+00	4.24E+00	3.43E-03
2.91E-01	3.07E+00	2.40E+00	7.46E-01	5.03E+00	0.00E+00	1.21E+00	4.24E+00	3.60E-03
3.03E-01	3.04E+00	2.30E+00	7.58E-01	4.97E+00	0.00E+00	1.22E+00	4.23E+00	3.77E-03
3.14E-01	3.00E+00	2.25E+00	7.69E-01	4.90E+00	0.00E+00	1.24E+00	4.23E+00	3.94E-03
3.26E-01	2.98E+00	2.25E+00	7.81E-01	4.84E+00	0.00E+00			4.11E-03
3.38E-01	2.94E+00	2.26E+00	7.93E-01	4.77E+00	0.00E+00			
3.49E-01	2.87E+00	2.36E+00	8.04E-01	4.70E+00	0.00E+00			
3.61E-01	2.89E+00	2.59E+00	8.16E-01	4.65E+00	0.00E+00			
3.73E-01	2.96E+00	2.85E+00	8.28E-01	4.62E+00	0.00E+00			
3.84E-01	3.13E+00	3.08E+00	8.39E-01	4.59E+00	0.00E+00			
3.96E-01	3.57E+00	3.13E+00	8.51E-01	4.56E+00	0.00E+00			
4.08E-01	3.92E+00	3.17E+00	8.63E-01	4.53E+00	0.00E+00			
4.19E-01	4.12E+00	3.16E+00	8.74E-01	4.50E+00	0.00E+00			
4.31E-01	4.31E+00	3.14E+00	8.86E-01	4.47E+00	0.00E+00			
4.43E-01	4.59E+00	3.15E+00	8.98E-01	4.46E+00	0.00E+00			
4.54E-01	4.94E+00	3.11E+00	9.09E-01	4.45E+00	0.00E+00			
4.66E-01	5.45E+00	2.85E+00	9.21E-01	4.43E+00	0.00E+00			
4.78E-01	5.52E+00	2.39E+00	9.33E-01	4.42E+00	0.00E+00			
4.89E-01	5.65E+00	1.92E+00	9.44E-01	4.41E+00	0.00E+00			
5.01E-01	5.62E+00	1.56E+00	9.56E-01	4.39E+00	1.27E-04			
5.13E-01	5.49E+00	1.28E+00	9.68E-01	4.38E+00	2.87E-04			
5.24E-01	5.31E+00	1.18E+00	9.79E-01	4.37E+00	4.48E-04			
			9.91E-01	4.35E+00	6.11E-04			
