# Supplementary material for: Tunable unidirectional nonlinear emission from transition-metal-dichalcogenide metasurfaces
Source: Nat Commun. 2021 Sep 22;12:5597. doi: 10.1038/s41467-021-25717-x (PMC8458373; doi:10.1038/s41467-021-25717-x)
Supplement: Supplementary file 2 — Description of Additional Supplementary Files [file 41467_2021_25717_MOESM2_ESM.pdf]

Description of Additional Supplementary Files

Supplementary Data 1: Description

Refractive index data used in the numerical simulations
